# Effects of Different Levels of Methionine in Liquid and Powder Forms on Growth Performance, Blood Parameters, and Carcass Traits of Broiler Chickens

**DOI:** 10.1002/vms3.71112

**Published:** 2026-07-20

**Authors:** Parvin Khosravi, Fatemeh Izadi Yazdanabadi, Hossein Sabzekar, Mahdi Rahmatinezhad, Saghar Zohouri, Amirhossein Hadiniya, Behnam Alizadeh

**Affiliations:** ^1^ Department of Animal Science Faculty of Agriculture Sari Agricultural Sciences and Natural Resources University Sari Iran; ^2^ Department of Animal Science Faculty of Agriculture University of Tabriz Tabriz Iran; ^3^ Department of Animal Science Faculty of Agriculture University of Zabol Zabol Iran; ^4^ Faculty of Veterinary Medicine University of Zabol Zabol Iran; ^5^ Department of Animal Science Faculty of Agriculture University of Birjand Birjand Iran

**Keywords:** blood biochemistry, broiler chickens, intestinal morphology, methionine sources, performance

## Abstract

Methionine is a key limiting amino acid in poultry nutrition and plays an essential role in growth, metabolism, and antioxidant defence. This study aimed to evaluate the effects of different sources and combinations of methionine on performance, carcass traits, intestinal morphology, and blood biochemical parameters in broiler chickens. A total of 300 one‐day‐old male broiler chicks were randomly assigned to six dietary treatments with five replicates of 10 birds each. The treatments included a control diet without methionine (60‐MET), 100% powder methionine (CONT), 100% liquid methionine (100‐LM), and three combinations of powder and liquid methionine (50‐LPM, 25‐LPM, and 75‐LPM). Body weight gain and feed conversion ratio were significantly improved in all methionine‐supplemented groups, particularly in 100‐LM and 50‐LPM (*p* < 0.05) compared with the 60‐MET group. Carcass yield did not differ significantly, but thigh and breast percentages increased in supplemented groups (*p* = 0.003 and *p* = 0.001, respectively) compared with the 60‐MET group. Intestinal morphology analysis showed a significant increase in villus height with methionine supplementation (*p* = 0.001), while crypt depth and muscular thickness remained unchanged. Blood biochemical analysis showed that methionine supplementation significantly reduced serum urea, uric acid, AST, ALT, and MDA levels (*p* < 0.05), while increasing albumin, total protein, and total antioxidant capacity (*p* < 0.05) compared with the 60‐MET group. In conclusion, both powder and liquid methionine sources, especially in the 100‐LM and 50‐LPM treatments, had positive effects on performance, gut morphology, and physiological health in broilers, indicating the efficiency of liquid form.

## Graphical abstract

Broiler chickens fed diets with liquid or combined methionine sources (especially 100‐LM and 50‐LPM) showed improved growth, feed efficiency, breast yield, villus height, and antioxidant status, with reduced serum urea, uric acid, and liver enzymes—demonstrating superior efficiency of liquid methionine supplementation.

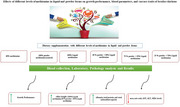

## 1 Introduction

Over the last 60 years, the poultry industry has made significant progress. Poultry meat is a high‐quality source of animal protein and plays an important role in supporting human nutrition and health (Oni et al. 2024). Compared to other meat industries, poultry meat production has achieved the most success. During this time, broiler production standards have improved, and today, male broilers can reach a body weight (BW) of 2.6 kg in 33–35 days (Ravindran and Abdollahi 2021).

Nutrition plays a vital role in improving the growth and growth performance of broiler chickens. Effective use of available crystalline amino acids (AAs) is essential for promoting optimal growth, lowering production costs, and minimizing the environmental footprint of commercial broiler chicks (Asasi et al. 2023). Broiler chicks diets need to be supplemented with synthetic sources of methionine (Met), such as DL‐Methionine (a mixture of D‐ And L‐ isomers) or DL‐2‐hydroxy‐4‐(methylthio)‐butanoic acid (DL‐HMTBA) (Gong et al. 2023). Met is the first limiting AA in broiler diets based on corn and soybean meal. It plays a vital role in broiler chickens, particularly in feather development and protein synthesis (Uddin et al. 2022).

Met can also be broken down into cysteine through the transmethylation–transsulfuration pathway and produces glutathione (GSH) that helps reduce the harmful effects of reactive oxygen species (ROS) (Jankowski et al. 2021). Therefore, a higher intake of Met or total sulphur amino acids (TSAA) is necessary to improve the performance of broiler chickens (Pacheco et al. 2018).

It has been reported that Met has the positive effects on growth performance (Gao et al. 2019; Rehman et al. 2019), carcass traits (Barido et al. 2019; Elsharkawy et al. 2021), and antioxidant enzymes (Kachungwa Lugata et al. 2022).

Biological availability (bioavailability) is the portion of a nutrient that the body can absorb and use (Littell et al. 1995). Met bioavailability is affected by its source, chemical form, other dietary components, and gut metabolism. Low‐quality diets and nutritional imbalances can reduce Met availability, highlighting the importance of proper nutrition in poultry (Ghazaghi et al. 2024). DL‐Met, a highly purified powder (99% purity), is the most common form of Met supplementation and contains equal proportions of l‐ and d‐isomers. The l‐isomer is considered the biologically active form, as it is the one incorporated into muscle proteins and enzymes (Sahebi‐Ala et al. 2021).

Thus, considering the essential role of Met in broiler nutrition and the differences in its bioavailability depending on the source and form, further studies are needed to compare the effects of different Met forms on broiler performance. While DL‐Met in powder form is commonly used, liquid forms may have better bioavailability under feeding conditions. However, limited studies have compared the effects of different levels and physical forms (liquid vs. powder) of Met on the growth performance, blood biochemical parameters, and carcass traits of broiler chickens. This study aims to evaluate and compare the effects of different levels of Met in liquid and powder forms on the performance, physiological responses, and carcass traits of broilers.

## 2 Materials and Methods

### 2.1 Animals

This study was carried out in accordance with the guidelines approved by the Animal Care Committee of the university of Tabriz, Iran. A total of 300 one‐day‐old male Ross 308 broiler chicks, with an average initial weight of 44 ± 2 g, were obtained from a local hatchery. The chicks were kept under a lighting program of 23 h of light and 1 h of darkness and housed in pens (1 × 1 m^2^) lined with fresh wood shavings. They had free access to both feed and water. The diets, based on corn and soybean meal, were formulated according to the nutritional requirements specified in the Ross management guide (Aviagen, 2014) (Table 1). The feeding program consisted of three phases: starter (1–10 days), grower (11–24 days), and finisher (25–42 days).

**TABLE 1:** Composition of diets.

Ingredients (g/kg)	Starter	Grower	Finisher
Corn	553.6	620.6	657.8
Soybean meal	392	327	290
Vegetable oil	10	12	15
Di‐calcium phosphate	16	13.5	11.5
Calcium carbonate	14	13.5	13
Mineral mixture^a^	2.5	2.5	2.5
Vitamin mixture^b^	2.5	2.5	2.5
Dl‐methionine	2.1	1.8	1.6
l‐Lysine	2.2	2	1.8
l‐Threonine	1.1	0.9	0.8
Salt	2.3	2.1	1.9
Sodium bicarbonate	1.7	1.6	1.6
**Nutrients**			
ME (Mcal/kg)	2850	2950	3000
Crude protein (%)	22	19.5	18.5
Ca (%)	0.95	0.87	0.79
Available phosphorous (%)	0.47	0.43	0.39
K (%)	0.85	0.75	0.70
Na (%)	0.17	0.16	0.16
Cl (%)	0.17	0.16	0.16
Met (%)	0.51	0.46	0.43
Met + Cys (%)	0.84	0.77	0.72
Lys (%)	1.27	1.12	1.02
Arg (%)	1.35	1.2	1.1
Thr (%)	0.82	0.72	0.67
Try (%)	0.23	0.2	0.19
Crude fibre (%)	3.8	3.4	3.2
Ether extract (%)	3.1	3.5	4
Choline	1.7	1.6	1.6
Linoleic acid (%)	1.5	1.7	1.9

^a^ Mineral premix provided per kilogram of diet: Mn (MN3O4), 120 mg; Zn (ZnSO4·H2O), 102 mg; Fe (FeSO4·5H2O), 40 mg; Cu (CuSO4·5H2O), 10 mg; I (ca (Io3)2, X H2O), 1.5 mg; and Se (Na2seo3), 0.35 mg.

^b^ Vitamin premix provided per kilogram of diet: vitamin A (retinyl acetate), 12,000 IU; cholecalciferol, 4500 IU; vitamin E (DL‐α‐tocopheryl acetate), 62.5 IU; vitamin K (menadione sodium bisulphite), 3 mg; thiamine, 3 mg; riboflavin, 6.6 mg; nicotin amide, 55 mg; calcium pantothenate, 20 mg; pyridoxine, 5 mg; folic acid, 1.92 mg; biotin, 0.20 mg; vitamin B12, 0.016 mg; choline (choline chloride, 60 %), 500 mg; and Antioxidant, 150 g.

In this study, the broiler chicks were divided into six groups, each consisting of five replicates with 10 chicks per replicate. The groups received diets containing different forms of Met as follows: a control group without supplementation (60‐MET), 100% Met in powder form (CONT), 100% Met in liquid form (100‐LM), 50% Met in liquid form and 50% in powder form (50‐LPM), 25% Met in liquid form and 75% in powder form (25‐LPM), and 75% Met in liquid form and 25% in powder form (75‐LPM). Liquid and powder forms were prepared from Adisseo Company (France) and CJ company (South Korea), respectively. Both were added into feed.

At the end of the trial, the broiler chicks were weighed following a 3‐h fasting period. Growth performance parameters, including BW, feed intake (FI), and feed conversion ratio (FCR), were measured. Mortality was monitored and recorded on a daily basis.

### 2.2 Carcass Traits

At 42 days of age, three chickens from each replicate were randomly selected, weighed, and humanely slaughtered for carcass evaluation. After complete bleeding, the birds were de‐feathered and eviscerated, and their hot carcass weights were recorded.

### 2.3 Blood Parameters

On day 42, two birds from each pen were randomly selected, individually weighed, and euthanized using electrical stunning followed by exsanguination. Blood samples were collected from each bird into 5 mL non‐heparinized tubes, centrifuged at 5000 rpm for 10 min, and transported on ice to the private Pathology Laboratory. The samples were analyzed using the enzyme‐linked immunosorbent assay (ELISA) kits from Pars Azmoon Company (Tehran, Iran), following the manufacturer's instructions.

### 2.4 Antioxidant Parameters

The serum samples were collected for further evaluation of antioxidant parameters. Total antioxidant capacity (TAC) was measured following the previously established method (Erel 2004). In this method, antioxidants in the serum reduce the dark blue‐green ABTS radical (2,2'‐azino‐bis [3‐ethylbenzothiazoline‐6‐sulfonic acid]) to a colourless form. Change in absorbance was measured using a spectrophotometer at a wavelength between 660 and 670 nm. Malondialdehyde (MDA) levels were assessed based on an earlier described method (Ohkawa et al. 1979). MDA reacts with deoxyadenosine and deoxyguanosine in DNA, forming MDA–DNA complexes. In the TBARS (thiobarbituric acid reactive substances) assay, MDA forms a fluorescent red derivative upon reacting with thiobarbituric acid, which was measured spectrophotometrically at 532 nm.

### 2.5 Intestinal Morphometric Parameters

The small and large intestines of slaughtered birds were removed to measure their weights and lengths. After dissection, the caecal tonsils, bursa of Fabricius, and small intestine were excised, and 5 cm segments of the jejunum were collected. The jejunum from the distal duodenal loop to Meckel's diverticulum was considered (Murakami et al. 2007). Intestinal samples were fixed in 10% buffered formalin, embedded in paraffin, and stained using the haematoxylin and eosin (H&E) method. Measured morphometric parameters included villus height, width, crypt depth, and muscularis externa. Villus height was measured from the tip to the base at the crypt junction, and crypt depth was measured from the base of the crypt to the transition zone between crypt and villus. Villus surface area was calculated using the formula: (2π) × (villus width/2) × villus length (Ashraf et al. 2013).

### 2.6 Data Analysis

The collected data were analyzed using a completely randomized design. Group means were compared using Duncan's multiple range (DMR) test using the SAS Software (version 13.1). A significance level of *p* < 0.05 was considered statistically significant.

## 3 Results

### 3.1 Growth Performance

Table 2 presents the effects of different Met sources and combinations on the growth performance of broiler chickens. There were no statistically significant differences in FI (*p* = 0.075), although the 50‐LPM and 75‐LPM groups showed numerically higher intake values. Birds supplemented with Met in all experimental groups (100‐LM, 50‐LPM, 25‐LPM, and 75‐LPM) had significantly higher BW compared to the 60‐MET group (*p* = 0.037). The 50‐LPM group showed the highest average BW. FCR improved significantly in all Met‐supplemented groups compared to the 60‐MET group (*p* = 0.007), with the lowest FCR observed in the 100‐LM group. Mortality rate was significantly reduced in the 25‐LPM group (*p* = 0.001), while other groups showed no significant differences from the control. The dietary Met supplementation, especially in combined liquid and powder forms, positively affected broiler growth performance and survival.

**TABLE 2:** The effects of different sources of methionine on the growth performance of broiler chicks.

Treatments	Feed intake (g)	Body weight (g)	FCR	Mortality (%)
60‐MET	4207 ± 166.53	2115.83 ± 192.53b	2.00 ± 0.25a	7.69a
CONT	3966 ± 189.99	2332.50 ± 110.48a	1.70 ± 0.13b	6.33a
100‐LM	3925 ± 164.91	2346.33 ± 188.42a	1.68 ± 0.15b	5.13ab
50‐LPM	4115 ± 200.40	2413.33 ± 160.20a	1.71 ± 0.14b	6.41a
25‐LPM	4003 ± 124.06	2370.33 ± 84.83a	1.69 ± 0.07b	2.56b
75‐LPM	4104 ± 187.87	2364.16 ± 169.15a	1.74 ± 0.13b	7.69a
SEM	31.49	29.07	0.03	
*p* values	0.075	0.037	0.007	0.001

*Note*: Different letters (a, b) show significant differences between the groups.

### 3.2 Carcass Traits

Tables 3 and 4 present the effects of different Met sources and combinations on carcass traits, immune organs, and intestinal lengths of broiler chickens at 42 days of age. Carcass yield did not differ significantly among treatments (*p* = 0.645), but all Met‐supplemented groups showed numerically higher values than the control. Thigh percentage increased significantly in all supplemented groups compared to the 60‐MET (*p* = 0.003), with the highest values observed in 25‐LPM and 75‐LPM groups. Breast muscle percentage was also significantly improved by Met supplementation (*p* = 0.001), especially in birds fed 50‐LPM and 100‐PM diets. Heart weight was significantly lower in all Met‐fed groups compared to 60‐MET (*p* = 0.008), with the smallest values recorded in the 100‐PM group.

**TABLE 3:** The effects of different sources of methionine on carcass traits of broiler chicks.

Treatments	Carcass (%)	Thigh (%)	Breast (%)	Liver (%)	Gizzard (%)	Pancreas (%)	Heart (%)
60‐MET	78.18 ± 1.33	21.13 ± 1.42b	26.77 ± 1.72b	0.04 ± 0.006	0.019 ± 0.003	0.003 ± 0.0007	0.007 ± 0.001a
CONT	79.71 ± 3.54	22.41 ± 2.01a	31.47 ± 1.65a	0.036 ± 0.008	0.017 ± 0.001	0.002 ± 0.0004	0.004 ± 0.0006b
100‐LM	78.60 ± 2.01	23.60 ± 1.26a	31.08 ± 1.90a	0.033 ± 0.008	0.017 ± 0.002	0.003 ± 0.0006	0.005 ± 0.0009b
50‐LPM	78.18 ± 1.33	23.07 ± 2.54a	31.71 ± 2.61a	0.04 ± 0.006	0.016 ± 0.003	0.002 ± 0.0003	0.005 ± 0.001b
25‐LPM	77.90 ± 1.35	24.00 ± 1.16a	31.05 ± 1.32a	0.036 ± 0.008	0.018 ± 0.004	0.002 ± 0.0005	0.006 ± 0.003b
75‐LPM	78.80 ± 2.23	24.05 ± 2.04a	30.69 ± 1.96a	0.039 ± 0.005	0.017 ± 0.005	0.002 ± 0.0005	0.005 ± 0.008b
SEM	0.44	0.40	0.57	0.001	0.0005	0.0001	0.0002
*p* values	0.645	0.003	0.001	0.544	0.270	0.160	0.008

*Note*: Different letters (a, b) show significant differences between the groups. The data were reported as percentage of carcass weight.

**TABLE 4:** The effects of different sources of methionine on immune organs and intestinal lengths.

Treatments	Thymus (%)	Spleen (%)	Bursa (%)	Duodenum (cm)	Jejunum (cm)	Ileum (cm)
60‐MET	0.003 ± 0.001	0.001 ± 0.0001	0.003 ± 0.0007	31.41 ± 1.96	83.50 ± 7.79	71.08 ± 6.97
CONT	0.003 ± 0.002	0.001 ± 0.0007	0.002 ± 0.0004	31.30 ± 1.64	87.00 ± 2.13	72.60 ± 4.62
100‐LM	0.003 ± 0.001	0.001 ± 0.0002	0.003 ± 0.0006	31.33 ± 1.96	85.66 ± 5.99	78.00 ± 5.72
50‐LPM	0.003 ± 0.002	0.0009 ± 0.0001	0.002 ± 0.0003	31.41 ± 1.67	83.83 ± 5.77	72.83 ± 4.21
25‐LPM	0.003 ± 0.002	0.009 ± 0.0004	0.002 ± 0.0005	31.59 ± 1.21	84.71 ± 3.29	74.45 ± 3.56
75‐LPM	0.003 ± 0.005	0.001± 0.0005	0.002 ± 0.0005	31.41 ± 1.96	86.32 ± 4.25	75.63 ± 3.96
SEM	0.003	0.0009	0.0001	1.35	3.71	3.48
*p* values	0.805	0.081	0.612	0.481	0.662	0.361

There were no significant differences among treatments for liver (*p* = 0.544), gizzard (*p* = 0.270), and pancreas (*p* = 0.160) weights, as well as for the relative weights of the thymus (*p* = 0.805), spleen (*p* = 0.081), and bursa of Fabricius (*p* = 0.612). Additionally, Met source did not significantly affect the lengths of the duodenum (*p* = 0.481), jejunum (*p* = 0.662), or ileum (*p* = 0.361). These findings suggest that while Met supplementation, especially in combined or pure forms, improves carcass characteristics such as thigh and breast yield, it has no adverse effects on organ development or intestinal length.

### 3.3 Intestinal Morphology

**FIGURE 1:**
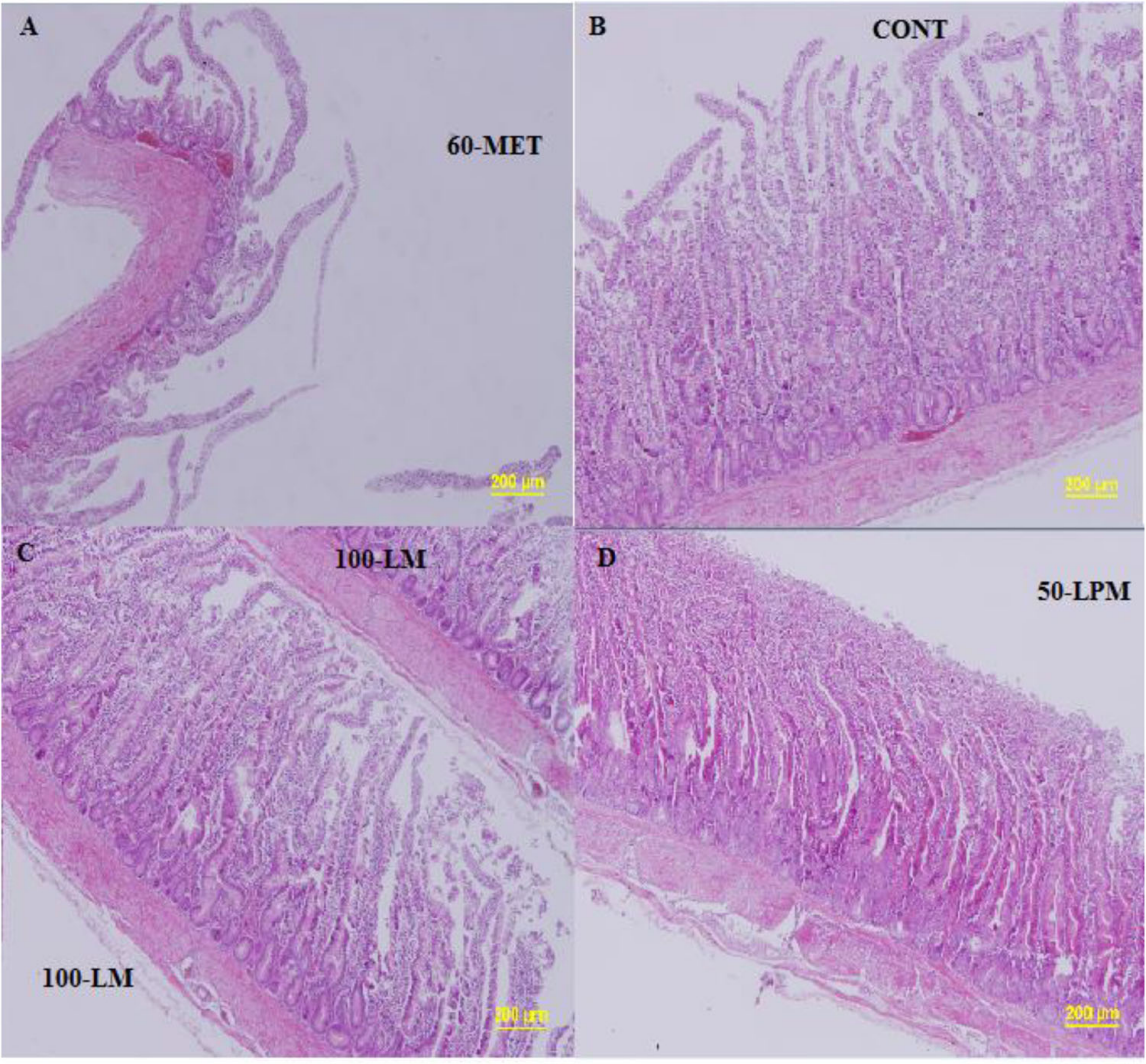
Pathology images for intestinal morphology. The results show an improvement in the supplemented broiler chicks. (A) Diet providing 60 % of the broiler’s methionine requirement, (B) Diet containing 100% methionine in powder form (CONT), (C) Diet containing 100% methionine in liquid form (100‐LM), (D) Diet containing 50% methionine in liquid form and 50% in powder form (50‐LPM).

Table 5 presents the effects of different Met sources and combinations on intestinal morphology of broiler chickens. Villus height was significantly affected by Met source (*p* = 0.001). The 60‐MET group showed the shortest villi (846.10 ± 152.20 µm), while all Met‐supplemented groups showed significantly taller villi, with the highest height recorded in the 100‐LM group (640.10 ± 175.20 µm). In contrast, villus width (*p* = 0.074), crypt depth (*p* = 0.143), and muscular thickness (*p* = 0.101) were not significantly affected by treatment, although some numerical variations were observed. Figure 1 confirm the results and shows an improvement in the supplemented broiler chicks. As the results show, in the control group (Figure 1B), the intestinal villi are long and well organized, the crypts are healthy and regular, and there is no evidence of inflammation or necrosis. The tissue shows a healthy and normal intestine in the chicks. In the 60% Met group (Figure 1A), the villi are slightly shorter and thicker than the control, there is a slight increase in intercellular space, and some blood spots are observed. In the 100‐LM and 50‐LPM groups, the condition of the villi and crypts is completely normal, indicating healthy intestinal tissue.

**TABLE 5:** The effects of different sources of methionine on intestinal morphology of broiler chicks.

Treatments (µm)	Villus height (µm)	Villus width (µm)	Crypt depth (µm)	Muscular (µm)
60‐MET	646.10 ± 152.20b	111.30 ± 28.54b	142.24 ± 28.23	207.60 ± 53.20
CONT	840.10 ± 175.20a	137.60 ± 25.42a	153.70 ± 51.97	182.44 ± 66.70
100‐LM	780.90 ± 117.80a	139.53 ± 31.42a	165.80 ± 40.97	182.07 ± 29.70
50‐LPM	749.60 ± 163.91a	131.57 ± 30.20a	151.80 ± 50.64	176.23 ± 46.22
25‐LPM	780.90 ± 120.80a	130.57 ± 34.83a	137.89 ± 42.13	181.23 ± 41.23
75‐LPM	752.10 ± 135.60a	134.16 ± 29.15a	146.52 ± 32.56	189.23 ± 26.32
SEM	16.23	3.00	3.60	4.76
*p* values	0.001	0.074	0.143	0.101

*Note*: Different letters (a, b) show significant differences between the groups.

### 3.4 Blood Parameters

Tables 6 and 7 summarize the effects of different Met sources on a range of blood biochemical parameters in broiler chicks. Birds in the 60‐MET group (CONT) showed significantly higher levels of urea (8.58 mg/dL), uric acid (5.67 mg/dL), AST (148.38 U/L), ALT (9.53 U/L), and MDA (1.25 mg/dL) compared to the supplemented groups (*p* < 0.05). It shows an increase in protein catabolism, liver stress, and oxidative damage. In contrast, all Met‐supplemented treatments showed significantly improved levels of albumin (2.90–3.90 mg/dL), total protein (2.45–3.85 mg/dL), and TAC (TAC; 1.61–1.91 mg/dL), with the highest values observed in the 50‐LPM and 100‐PM groups (*p* < 0.05). No significant differences were observed in glucose, cholesterol, triglycerides, creatinine, HDL, or LDL levels (*p* > 0.05).

**TABLE 6:** The effects of different sources of methionine on blood biochemical parameters (mg/dL) of broiler chicks.

Treatments	Glucose	Cholesterol	Triglycerides	Urea	Albumin	Total protein	Uric acid
60‐MET	206.31 ± 24.66	135.01 ± 29.21	33.71 ± 9.72	8.58 ± 0.46a	2.17 ± 0.63b	2.87 ± 0.57b	5.67 ± 1.16a
CONT	211.80 ± 38.22	110.11 ± 32.21	27.87 ± 10.23	7.02 ± 0.38b	3.90 ± 0.71a	3.53 ± 0.54a	3.89 ± 1.23b
100‐LM	223.32 ± 54.41	139.45 ± 30.21	29.03 ± 8.85	7.14 ± 0.38b	3.01 ± 0.82a	3.57 ± 0.46a	3.06 ± 0.97b
50‐LPM	204.84 ± 17.21	131.44 ± 28.14	41.21 ± 9.62	6.69 ± 0.36b	3.34 ± 0.73a	3.85 ± 0.53a	3.74 ± 1.02b
25‐LPM	211.30 ± 18.35	129.10 ± 15.23	36.23 ± 8.63	6.85 ± 0.32b	3.11 ± 0.84a	3.71 ± 0.45a	3.36 ± 1.10b
75‐LPM	216.30 ± 21.23	124.25 ± 20.24	30.69 ± 10.10	6.32 ± 0.45b	3.26 ± 0.25a	3.45 ± 0.45a	3.25 ± 1.02b
SEM	6.86	5.91	11.47	0.51	0.75	0.71	0.87
*p* values	0.796	0.338	0.131	0.031	0.027	0.023	0.041

*Note*: Different letters (a, b, c) show significant differences between the groups (*P* < 0.05).

**TABLE 7:** The effects of different sources of methionine on blood biochemical parameters (mg/dL) of broiler chicks.

Treatments	Creatine	HDL	LDL	AST	ALT	TAC	MDA
60‐MET	0.218 ± 0.01	55.70 ± 4.63	41.66 ± 8.72	148.38 ± 15.23a	9.53 ± 1.96a	1.37 ± 0.17c	1.25 ± 0.14a
CONT	0.206 ± 0.02	50.65 ± 5.85	42.83 ± 9.23	128.63 ± 18.23b	7.53 ± 1.36b	1.91 ± 0.37a	1.04 ± 0.06b
100‐LM	0.221 ± 0.02	54.91 ± 6.23	44.50 ± 8.96	105.44 ± 14.23c	6.70 ± 1.37b	1.61 ± 0.20b	1.10 ± 0.09b
50‐LPM	0.227 ± 0.02	52.41 ± 7.21	48.15 ± 6.36	129.32 ± 10.65b	6.20 ± 2.56b	1.91 ± 0.23a	1.04 ± 0.11b
25‐LPM	0.218 ± 0.01	53.10 ± 5.63	41.05 ± 7.25	126.32 ± 12.36b	6.48 ± 1.21b	1.67 ± 0.25b	1.08 ± 0.08b
75‐LPM	0.205 ± 0.02	50.41 ± 6.32	40.25 ± 8.25	107.81 ± 13.36c	6.66 ± 1.14b	1.82 ± 0.15a	1.06 ± 0.09b
SEM	0.005	2.44	2.77	11.72	0.52	0.10	0.04
*p* values	0.595	0.107	0.525	0.012	0.030	0.021	0.015

*Note*: Different letters (a, b, c) show significant differences between the groups (*P* < 0.05).

## 4 Discussion

Birds supplemented with Met in all experimental groups had significantly higher BW and lower FCR compared to the 60‐MET group. The results are consistent with those reported by Hayat et al. (2015) who reported that Met sources could significantly have positive effects on the growth performance of broiler chicks. The results are also in agreement with others that reported sources of Met along with cysteine had positive effects on the growth performance of broiler chicks (Rehman et al. 2019). Met‐deficient diets negatively impacted broiler growth during the entire experiment, highlighting the need to provide sufficient Met in their feed to ensure optimal growth and performance. Met is essential to the synthesis of proteins and organs, as shown for thigh and breast. It also increases villus width and length that helps to more absorption of nutrients that are utilized for growth performance and results in better FCR. In addition, it is essential to maintain liver health, as shown by liver enzymes and antioxidant parameters. Both powder and liquid forms of Met showed similar effects on broiler performance. This similarity may be because both forms provide bioavailable Met that meets the birds’ nutritional needs equally well. Additionally, the absorption and metabolism of Met from either form appear to be efficient, allowing the birds to utilize the AA effectively for growth and development. Therefore, either form can be used as a reliable Met source in broiler diets without compromising performance.

Met supplementation could significantly increase thigh and breast weight, but it did not have significant effects on other organs. The results are consistent with other studies that reported the positive effects of Met on thigh and breast weights (Elsharkawy et al. 2021; Wu et al. 2021). Ojano‐Dirain and Waldroup (2002) found that broilers fed higher levels of Met than the National Rese (NRC) recommendation had better dressing percentage and more breast meat compared to those given only the NRC level. Generally, the significant effects of Met supplementation on thigh and breast percentages, but not on other carcass parts or organs, may be due to the fact that these muscles grow rapidly and require more AAs such as Met for protein synthesis. Met plays a key role in muscle development, especially in fast‐growing tissues such as the breast and thigh muscles in broilers (Herich et al. 2025; Macelline et al. 2021). Other organs, such as the liver, heart, and spleen, may not be as sensitive to Met levels because their growth is less directly tied to protein deposition. This is also supported by the intestinal morphology results, where improved villus height in supplemented groups suggests better nutrient absorption, possibly promoting muscle growth more than organ enlargement.

The results showed that Met supplementation significantly increased villus width and height. The results concur with other studies that reported that Met supplementation increased villus width and height in broiler chicks (Miao et al. 2021). Another study also reported that Met supplementation could significantly improve intestinal morphology (de Moraes et al. 2021). Wang et al. (2022) showed that lack of enough Met reduced the growth and size of intestinal organoids. They also found that using a Met helped intestinal stem cells grow again. Different Met sources had significant effects on villus height but not on villus width. Diets containing Met, in powder, liquid, or mixed forms, significantly increased villus height compared to the 60‐MET group, which may be due to improved protein synthesis and intestinal cell turnover supported by adequate Met. Taller villi provide a larger surface area for nutrient absorption, which can support better growth performance. However, crypt depth did not show significant changes, likely because width is less sensitive to short‐term dietary changes and may be more influenced by structural or genetic factors rather than nutrient availability.

The results showed that Met supplementation could increase the concentration of albumin and protein while decreasing urea and uric acid compared to 60‐MET group, which is parallel with other studies for the effects of Met on serum albumin (Park and Kim 2019). Met helps improve protein synthesis and nitrogen retention in the body. When enough Met is available, the body can use AAs more efficiently to build proteins like albumin, instead of breaking them down that decreases the production of waste products such as urea and uric acid. This suggests better protein metabolism and less nitrogen loss in birds receiving adequate Met.

Met supplementation could decrease liver enzymes and improve antioxidant status in broiler chicks. It has been reported that Met has good potential to stimulate the antioxidant status of poultry (Kachungwa Lugata et al. 2022). The results are consistent with other studies for the effects of Met on antioxidant enzymes (J. Zeitz et al. 2018; J. O. Zeitz et al. 2020) and liver enzymes (Stojanović et al. 2018; Yang et al. 2017).

Met supplementation could decrease liver enzyme levels and improve antioxidant status by increasing TAC and decreasing MDA. These two outcomes are closely related, as lower liver enzyme levels suggest reduced liver stress that may result from increased antioxidant protection. Met plays a key role in GSH synthesis, a major antioxidant that helps protect liver cells from oxidative damage (Martínez et al. 2017). Therefore, better antioxidant defence likely contributes to improved liver health in broiler chicks.

## 5 Conclusion

This study showed that supplementing broiler diets with different sources and combinations of Met positively affects growth performance, carcass traits, intestinal morphology, and certain blood biochemical parameters. Birds fed with Met, especially in liquid or combined forms, showed improved BW gain, FCR, and increased breast and thigh yields compared to the 60‐MET group. Intestinal villus height was significantly increased in the supplemented groups that show an improved nutrient absorption capacity. Met supplementation also decreased levels of AST, ALT, and MDA while increased TAC, suggesting a protective effect on liver function and oxidative stress. These findings highlight the importance of adequate Met levels and implicates both liquid and mixed forms can be effectively used in broiler nutrition to support optimal health and productivity.

## Author Contributions

Fatemeh Izadi Yazdanabadi: writing – review and editing, writing – original draft, methodology, conceptualization, investigation, funding acquisition, resources. Amirhossein Hadiniya: investigation, funding acquisition, resources. Behnam Alizadeh: investigation, funding acquisition, resources. Saghar Zohouri: investigation, funding acquisition, visualization, resources. Parvin Khosravi: software, data curation, supervision, project administration, resources. Mehdi Rahmatinezhad: resources, funding acquisition. Hossein Sabzekar: data curation, software, formal analysis, methodology, funding acquisition, resources.

## Funding

The authors have nothing to report.

## Ethics Statement

All ethical considerations including utilizing animals were considered cautiously. The trial convention was affirmed by the animal welfare committee (which covered IACUC approval) of the Faculty of Veterinary Medicine, University of Tabriz, Tabriz, Iran. All applicable international, national, and/or institutional guidelines for the care and use of animals were followed. This case was managed in accordance with the ethical principles of veterinary clinical practice. Humane euthanasia was performed due to the grave prognosis, and the procedure complied with the ethical guidelines of the Hospital.

## Data Availability

The data that support the findings of this study are available from the corresponding author upon reasonable request.
